# Audio-visual source separation with localization and individual control

**DOI:** 10.1371/journal.pone.0321856

**Published:** 2025-05-23

**Authors:** Mohanaprasad Kothandaraman, Balakrishnan Ramalingam, Kai Sheng, Aman Verma, Utkarsh Dhagat, Pranav Parab, Siddhartha Mallavolu, Sankar Ganesh

**Affiliations:** 1 School of Electronics Engineering (SENSE), Vellore Institute of Technology, Chennai, India; 2 School of Computer Science and Engineering (SCOPE), Vellore Institute of Technology, Chennai, India; 3 Xidian University, Xi’an, China; UO: University of Okara, PAKISTAN

## Abstract

The growing reliance on video conferencing software brings significant benefits but also introduces challenges, particularly in managing audio quality. In multi-participant settings, ambient noise and interruptions can hinder speaker recognition and disrupt the flow of conversation. This work proposes an audio-visual source separation pipeline designed specifically for video conferencing and telepresence robots applications. The framework aims to isolate and enhance the speech of individual participants in noisy environments while enabling control over the volume of specific individuals captured in the video frame. The proposed pipeline comprises key components: a deep learning-based feature extractor for audio and video, an audio-guided visual attention mechanism, a module for background noise suppression and human voice separation, and Deep Multi-Resolution Network (DMRN) modules. For human voice separation, the DPRNN-TasNet, a robust deep neural network framework, is employed. Experimental results demonstrate that the methodology effectively isolates and amplifies individual participants’ speech, achieving a test accuracy of 71.88 % on both the AVE and Music 21 datasets.

## 1 Introduction

In today’s fast-paced world, video and audio conferencing applications play a pivotal role in connecting individuals and businesses across the globe. These applications have become essential tools for communication, collaboration, and productivity, enabling people to connect face-to-face regardless of their physical location. Whether used for virtual meetings, remote work, online education, or social interactions, video and audio conferencing applications have transformed the way we communicate and collaborate. While video and audio conferencing apps have revolutionized communication and collaboration, they do come with certain disadvantages. One common issue is the presence of background noise, especially when many individuals are interacting in a single video frame. This noise can be distracting and make it difficult for participants to hear each other clearly.

Noise removal is an essential stage in audio processing, following which individual human voices are isolated. Audio source separation is an essential method in signal processing that seeks to isolate distinct audio sources from a combined audio output. The isolation enables precise control over each source, which is crucial for improving audio quality by reducing background noise.By differentiating the required audio sources from the surrounding background noise,specific noise reduction techniques can be performed more efficiently, enhancing both the clarity and comprehensibility of the primary audio signals. To achieve these objectives, beamforming, source separations, adaptive filtering, and Deep Learning (DL) techniques are utilized [1–4].

Among them, the DL based techniques shows a better accuracy in audio source separation and noise cancellation. The literature presents a diverse range of methods based on above techniques for audio source separation that are specifically designed for various uses. In [5], Zhou *et al*. presented AVSBench, a benchmark for audio-visual segmentation that provides detailed annotations for sounding objects in audible videos at the pixel level. This study highlights the utilization of a temporal pixel-wise audio-visual interaction module to direct the visual segmentation process using audio semantics. Additionally, a regularization loss is employed to improve audio-visual correlation during the training phase. Forster *et al*. [6] implemented an unsupervised model-based deep learning approach for separating musical sources. In this method, each source is represented by a differentiable parametric source filter model. The approach involves training a neural network to restore the combined signal by approximating the characteristics of each source model using their fundamental frequencies. Tian *et al*. [7] introduced a method to localize audio-visual events in unrestricted films. They utilized an audio-guided visual attention mechanism, a Dual Multimodal Residual Network (DMRN), and an audio-visual distance learning network to enable accurate localization across different modalities. Stoller *et al*. [8] train the Wave-U-Net framework for complete audio source separation. The framework takes on the audio waveform as input and isolate distinct sources, such as vocals, drums, and bass, from a combined signal. In [9], David *et al*. suggested a strategy for tackling the source separation problem in the presence of numerous voice signals. The method uses automatic lipreading to distinguish an acoustic speech signal from other acoustic signals based on their coherence with the speaker’s lip movements. In another study, Gao *et al*. [10] utilize co-separation and localized object identification to visually direct audio source separation. This approach utilizes the complementary information in visual and auditory modalities to separate sound sources associated with distinct visual elements in a scene. The proposed scheme uses DL techniques to separate and identify sounds associated with individual objects in complicated contexts by using correlations between visual features and audio spectrograms. Li *et al*. [11] solve the audio-visual source association problem by comparing visual and auditory information. The proposed approach employs multimodal vibrato analysis to provide a comprehensive understanding of the relationship between visual and auditory performance cues, paving the way for improved musical interpretation and synchronization. Rahman *et al*. introduce a weakly supervised audio-visual sound source detection and separation method to localize and separate individual object sounds in the audio channel of a video [12]. Here, the authors developed an audio-visual co-segmentation network that learns both the visual and auditory characteristics of individual objects from videos labeled only with object labels. By jointly learning from both modalities, the network can effectively localize and separate sound sources in complex audiovisual scenes. Jie et.al developed unsupervised learning models for audio-visual localization and separation tasks [13]. The method employs low-rank modeling to capture background visual and audio information and sparsity to extract sparsely correlated components between the audio and visual modalities. Appearance and Motion network (AMnet) is proposed by Zhu and Esa to extract individual audio components from a mixture using video data from sound sources [14]. Leon *et al*. [15] developed a framework that integrates Deep Neural Networks (DNNs) and beamforming to provide efficient localization, separation, and reconstruction of sound sources utilizing arrays of microphones. This technology incorporates powerful machine learning techniques to enhance accuracy and decrease computing burden in intricate acoustic settings, establishing new standards for real-time audio processing. The authors use the self-supervised motion representation technique to train the proposed two-stage architecture to separate and localize sound sources in complex audio-visual scenes. Islam *et al*. [16] address the challenge of audio source separation for both known and unknown objects. They introduce a meta-consistency driven test-time adaptation strategy, enhancing model adaptability through a self-supervised audio-visual consistency objective. Zhang *et al*. [17] address the limitation of sound source separation models that only consider audio data by integrating a Dual-channel attention mechanism that leverages both audio and visual inputs. Their model, Audio-Visual separation integrating the Dual-channel Attention mechanism (AVDA), dynamically fuses audio and visual features to improve the quality of sound separation, and it shows significantly better performance on the MUSIC-21 dataset than previous models, as demonstrated by higher scores in sound distortion, interference, and artifact ratios. Long *et al*. [18] presents a method that utilizes a deep neural network (DNN) and a microphone array to accurately determine the location, separate, and reconstruct various sound sources. The study investigates a combined signal processing approach that combines beamforming techniques with deep neural networks (DNNs), demonstrating particular efficacy in circumstances where there are multiple sound sources that overlap. The technique improves precision and decreases computing burdens, confirmed through simulations and experimental configurations. The self supervised technique proposed by Yang *et al*. [19] enhances sound localization in films. The strategy aims to enhance the auditory aspect within a multimodal audio-visual learning framework. The authors introduce a novel approach that clusters audio attributes to generate pseudo-labels, which are subsequently employed to guide the training of the audio processing core. When utilized with MUSIC datasets [20], this method significantly enhances the precision of identifying sound sources by optimizing the integration and utilization of audio cues in the audio-visual correspondence learning process. Sanabria-Macias *et al*. [21] proposed an improved audiovisual tracking system that combines particle filters and probabilistic models to accurately determine the 3D position in intelligent environments. This is achieved by merging audio and video data. This system greatly enhances the precision of tracking in situations that are constantly changing and have obstructed views. It achieves this by employing a visual appearance model that takes into account the position of the subject’s lips, as well as an audio likelihood model that is based on a probabilistic variant of SRP-PHAT. A multimodal speaker diarization system is presented by Ahmad *et al*. [22], which utilizes a pre-trained SyncNet model to synchronize audio and visual data. This synchronization process improves the accuracy of speaker identification. This system utilizes Gaussian mixture model-based clustering to analyze synchronized audio-visual segments, resulting in lower diarization error rates compared to approaches that exclusively use audio. The technique exhibits significant enhancements in situations involving simultaneous speaking and dynamic interactions among participants. Liu *et al*. [23] propose a system for real-time speech separation that combines camera and microphone array sensors to improve speech quality in noisy conditions. By employing computer vision for speaker detection and beamforming for sound isolation, this approach greatly enhances the clarity and comprehensibility of speech. It efficiently decreases noise in both static and dynamic situations, showing promise for applications in assistive listening and machine language processing.

The aforementioned implementations have been utilized either for the purpose of segregating audio sources or for the purpose of determining the location of human audio sources. Previous studies on the subject have failed to develop a pipeline that allows for individual control over audio sources, including non-human sounds, after detecting them. This utility has the potential to be extremely useful in a wide range of fields, and this work tries to address that limitation by providing control of each source present in the video frame

The manuscript is structured as follows, In Sect [2]2, a thorough description of the proposed methodology is discussed. Sect [3]3 discusses about the dataset and experimental setup and presents the results of the experiments. Finally, Sect 4 provides a brief summary of the findings and identifies potential areas for future scope.

## 2 Materials and methods

**Fig 1:**
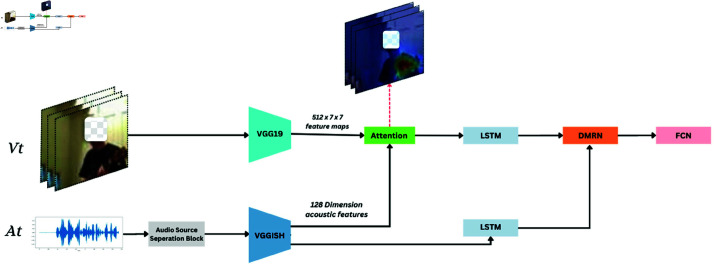
Block diagram of our proposed system.

The audio-visual source separation pipeline, illustrated in Fig 1, commences with specialized feature extractors for audio and video inputs that pre-process these streams for further thorough analysis. The video characteristics are extracted utilizing a Convolutional Neural Network (CNN), namely VGG19, which discerns vital spatial attributes including shapes, edges, and textures necessary for scene comprehension. Simultaneously, audio data undergoes preliminary processing in the audio source separation block, where ConvTasNet isolates human speech from ambient noise, and DPRNNTasNet performs monaural speech separation, enhancing speech clarity and intelligibility. Subsequent to this phase, the separated audio sources are input into VGGish, which excels at extracting intricate audio features that represent the spectral and temporal characteristics of the sounds. This guarantees that each audio piece is examined without disruption from overlapping noises, yielding a clearer and more distinct set of information for subsequent processing.

The elements from both the audio (VGGish) and video streams are subsequently integrated within an attention layer. This layer assesses the audio-influenced visual attributes, enhancing attention on video elements that align with prominent audio signals, thus maximizing the pertinence of the visual information concerning the audio input.

Convolutional Neural Networks (CNNs) are employed for their proficiency in extracting hierarchical spatial features from visual and auditory data, crucial for understanding complex images and sounds. LSTM networks are utilized to capture temporal relationships and sequence dynamics in data, essential for processing continuous time inputs like video frames and audio signals.Two LSTM networks are integrated into our framework. The first LSTM processes features extracted by VGGish, while the second analyzes sequential data from both modalities, as generated by the attention module. These networks adeptly capture and track the temporal dynamics within the video frames and the evolving auditory environments.

The outputs from the LSTM models, which now include integrated and augmented spatial and temporal information from video and audio data, are processed via the Dual Multimodal Residual Network (DMRN). This network utilizes a fusion layer to integrate the representations of both modalities. The resultant unified representation is subjected to further processing via LSTM to improve the audio signals, improving speech separation and reducing background noise, therefore illustrating the efficacy of the DMRN architecture.

In the final level of the model, a Fully Connected Neural Network (FCN) layer serves as the primary decision-making unit, utilizing sigmoid activation functions. This layer integrates multi-modal information from prior stages by a linear transformation, expressed as y=Wx+b, where *W* denotes the weight matrix and *b* signifies the bias vector. The sigmoid function is defined as $\sigma (z)=\frac{1}{1+e^{-z}}$.

Transforms these outputs into probabilities that signify the likelihood of specific events within the video frame. This transformation is essential as it offers a quantitative assessment of the model’s predictions, improving its capacity to precisely analyze intricate events from the combined audio visual data. The detail of each module and its functionality is described as follows.

### 2.1 VGG19 video feature extractor

The VGG19 model, pre-trained on the ImageNet database, is utilized for visual feature extraction from the visual inputs $V_{t}$. VGG19 is a deep convolutional neural network designed for the extraction of spatial elements, including edges, forms, and textures, which are essential for visual picture comprehension. The video input is processed at a rate of 24 RGB frames per second, with each frame transmitted through the network to produce feature maps measuring 512×7×7. These feature maps encapsulate elevated representations of visual content, including crucial spatial features for efficient alignment and integration with aural input in later processing phases.

### 2.2 Audio source separation

**Fig 2:**
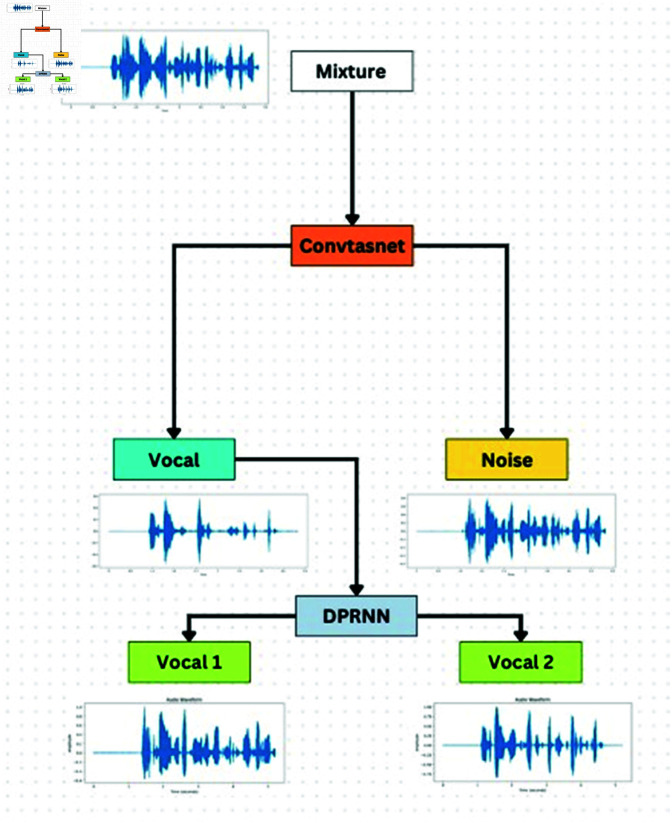
Audio source separation module.

**Fig 3:**
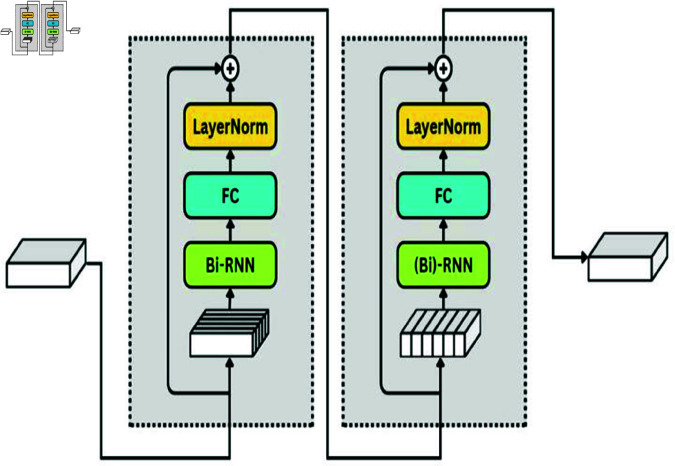
DPRNN-Tasnet module.

Audio source separation module as shown in Figs 2 and 3 process the audio signal separated from video streams. Audio source separation is a two-stage pipeline module that involves two key processes: separating noise sources and isolating human voices from the input audio signal. Noise source separation is a preliminary step in audio source separation that isolates noise from the audio and recovers the segment of the audio that predominantly consists of human voices to avoid interference from background noise during audio-visual event computation. Within the audio source separation pipeline, two models are employed consecutively to pre-process the audio input. At first, the Conv-TasNet [24] algorithm eliminates ambient noise from the audio stream. Afterwards, DPRNN-TasNet [25] further processes the cleaned audio to separate the voices of individual speakers. After the audio is produced, it can be used for feature extraction and further processing by the LSTM network to perform audio-visual event localization tasks. Employing sophisticated separation techniques is crucial to minimize noise and maximize useful signal in the audio component of the system. Conv-TasNet and DPRNN-TasNet are integral components, each contributing uniquely to the audio processing pipeline.

Conv-TasNet is used to separate the noise from the audio signal. It is a deep neural network architecture utilized for monaural speech separation, the process of isolating individual speakers from a mixed audio input that includes multiple speakers. Conv-TasNet’s architecture originates from the Time-domain Audio Separation Network (TasNet), comprising an encoder module, a separator module, and a decoder module. The encoder module transforms the input mixed audio stream into a series of high-level feature representations. The model consists of several 1D convolutional layers, followed by down sampling operation by using max-pooling. The encoder produces a low-dimensional representation of the input audio signal that captures the key elements necessary for separation. The separator module is in charge of isolating individual speakers from the mixed audio signal. It consists of a stack of several 1D convolutional layers with dilated convolutions to capture long-term temporal dependencies in the input signal. The separator module produces an output that consists of a collection of masks that designate the segments of the input signal attributed to each speaker.

Further, DPRNN-TasNet is used for monaural speech separation, which separates individual speakers from a mixed audio signal containing multiple speakers. DPRNN-TasNet is a neural network model that use a deep recurrent neural network (RNN) to predict the time-frequency masks for individual speakers in a mixed audio input. The masks are used on the mixed audio to extract individual audio signals for each speaker. The model utilizes the TasNet architecture, leveraging a convolutional neural network (CNN) for generating the initial time-frequency representations of the input audio signals. The CNN output is fed into a series of stacked recurrent layers to conduct the necessary time-domain processing for separating the mixed audio inputs into distinct speech signals. In DPRNN-TasNet, the recurrent layers are replaced with a Deep Recurrent Neural Network (RNN) that uses the Dual-Path Recurrent Neural Network (DPRNN) structure.

### 2.3 VGGish audio feature extractor

VGGish model, pre-trained on the extensive AudioSet dataset, is utilized for auditory feature extraction from the audio inputs *A*_*t*_. This model emphasizes the acquisition of essential auditory attributes, including rhythm, pitch, and timbre, which are vital for the analysis and synchronization of audio-visual events. Multiple VGGish models are employed to manage the complexity and diversity of audio signals, with each model addressing a specific audio source. This approach processes individual audio streams separately, ensuring customized feature extraction that enables precise alignment with the associated visual data. The VGGish model generates spectral and temporal audio features that improve the system’s capacity to localize and evaluate audio-visual events, even in difficult situations with overlapping or intricate sound environments.

### 2.4 Audio-guided visual attention

Audio-visual event localization network was utilized for localizing the audio sources from video frames. It takes input from both VGG19 and VGGish feature extractor and involves identifying the type and temporal boundaries of events in a video sequence using visual and auditory information generated by feature extractor $\omega_{m}$ and *A*_*t*_. The model integrates information from visual and auditory modalities using an attention mechanism to choose relevant visual elements based on the audio input. This method enables the model to focus on the most pertinent aspects of the visual data for each auditory input, hence enhancing the precision of event localization.

An essential part of integrating visual features with audio cues is the computation of the visual context vector, $\omega_{cd}$. An attention mechanism powers the computation, represented by the following equation:


$$\omega_{v_{C}}$$

In this case, the attention function that dynamically combines visual information weighted by related auditory inputs is represented by $\omega_{m_{C}}$.

The definition of the attention function is:


$$f_{\text{att}}(a_{t},v_{t})=\sum_{i=1}^{k}w_{i,t}v_{i,t}$$

According to their significance to the current audio input, each visual feature $v_{i,t}$ in this formulation is given an attention weight *w*_*i*,*t*_. The softmax function is used to create these weights, emphasizing the importance of each feature by normalizing the inputs into a probability distribution.

The attention weights *w*_*t*_ are calculated as follows:


$$w_{t}=\text{Softmax}(x_{t})$$

$$x_{t}=W_{f}\sigma (W_{v}U_{v}(v_{t})+W_{a}U_{a}(a_{t}))$$

This section incorporates the non-linear activation function σ, typically the hyperbolic tangent function, to add non-linearity to the learning process and enable the model to recognize more complex patterns. The transformation functions $U_{v}$ and *U*_*a*_ transform audio and visual inputs into a unified feature space that facilitates efficient integration.

### 2.5 LSTM

The LSTMs in the design are essential for capturing temporal dependencies in the visual and audio streams. The 512×7×7 spatial feature maps produced by VGG19 and enhanced by the attention mechanism are fed into an LSTM to acquire sequential patterns across frames. The 128-dimensional audio features collected by VGGish are similarly processed by an additional LSTM to encode temporal correlations within the auditory domain. These LSTMs function unidirectionally (forward), generating temporally coherent feature representations suitable for multimodal integration through the DMRN.

### 2.6 Dual multimodal residual network (fusion)

**Fig 4:**
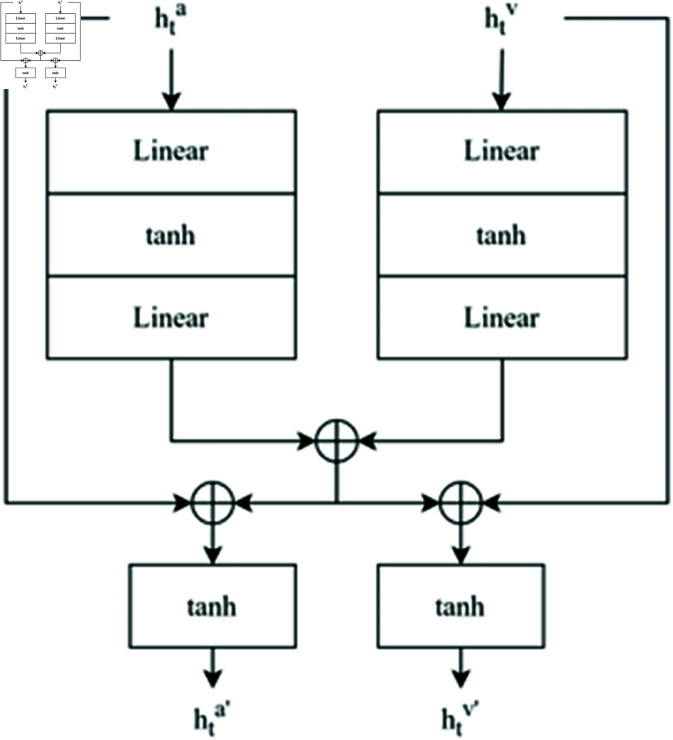
Dual multimodal residual network for audio-visual feature fusion.

The hidden states at time *t* for audio and visual inputs are denoted by $\sigma_{m_{F}}$ and $\sigma_{{cd}_{F}}$, respectively, and are obtained from distinct LSTM networks. These states encode the temporal dynamics of the corresponding modalities: visual aspects like forms and movements for $\sigma_{v_{P}}$, and audio features like rhythm and pitch for $\sigma_{m_{P}}$. After further processing through linear and tanh layers, these states enable synchronized and enhanced multimodal integration. Fusion layer uses the DMRN (Fig 4) which is a type of neural network architecture that is designed to process data from two different modalities, such as audio and visual inputs. It handles both audio and visual inputs simultaneously, allowing it to learn representations that capture the relationships between the two modalities. The DMRN architecture consists of two streams: one for processing audio inputs and another for processing visual inputs. Each stream contains several residual blocks that extract features from the respective modality. The outputs of these streams are then fused together in the network to allow for joint processing of the multimodal information.

$$h_{t}^{at}=\sigma (h_{t}^{a}+f(h_{t}^{a},h_{t}^{v}))$$ (1)

$$h_{t}^{vt}=\sigma (h_{t}^{v}+f(h_{t}^{a},h_{t}^{v}))$$ (2)

Here, $h_{t}^{a}t$ and $h_{t}^{v}t$ are the updated audio and visual features. represents an addition fusion function[1 , 2]. In our proposed framework, the DMRN network get the updated visual and audio features from the LSTM network and perform the fusion task.

### 2.7 Fully connected layer

The final stage of our model involves a decision-making process regulated by a fully connected neural network layer utilizing sigmoid activation function. This layer amalgamates multi-modal information from prior layers by a linear transformation, technically denoted as y=Wx+b, where *W* signifies the weight matrix and *b* denotes the bias vector. The sigmoid function $\sigma (z)=\frac{1}{1\;+\;e^{-z}}$ is then utilized on these linear outputs, transforming them into probabilities. These probabilities denote the chance of particular occurrences transpiring within the video clip. This output is essential, since it offers a quantitative assessment of the interactions between aural and visual input, hence augmenting the model’s ability to generate accurate and interpretable predictions.

The output of the fully connected layer comprises probabilities linked to certain audiovisual events. These probabilities allow the model to differentiate between various scenarios, such as recognizing specific noises associated with particular visual features or actions. This renders the model especially proficient in applications necessitating comprehensive knowledge and analysis of intricate occurrences, such as surveillance and multimedia content analysis. The preceding separation of audio sources enables the fully connected layer to process clearer, more distinct data, resulting in more accurate and dependable predictions.

## 3 Results

This section describes the experimental methods and results. The experiments were performed with AVE and music 21 dataset and its performance were evaluated with standard performance metrics

### 3.1 Dataset

AVE dataset is typically used for tasks such as sound source localization, audio-visual event detection, and multi-modal learning. It contains 4,143 videos in total, and it includes 28 different types of labels. These labels correspond to different types of audio-visual events that occur in the videos, such as “door knock,” “phone ringing,” “keyboard typing,” and others.

Music21 is a Python-based framework for computer-aided musicology that facilitates the analysis, research, and creation of music. It enables users to examine extensive music databases, provide musical examples, instruct in music theory, modify notation, and write music. The motto, “Listen Faster,” signifies the objective of allowing consumers to devote more time to appreciating music rather than engaging in arduous research. Music21 has been under development since 2008 and is grounded in academic traditions, primarily at MIT, where the “21” signifies its origins in the music department (Course 21). The toolbox is expanding and is extensively utilized in musicology, education, and composition.

### 3.2 Experimental settings

The experimental configuration,amalgamates visual and auditory modalities via an extensive pipeline that incorporates pre-trained CNNs, an audio-guided attention mechanism, and temporal modeling utilizing LSTMs, ultimately employing a Dual Multimodal Residual Network (DMRN) for fusion. For visual inputs $\sigma_{v_{F}}\leq \frac{1}{3}\delta_{1}$, each video segment is divided into 1-second intervals, from which 16 RGB frames are uniformly extracted. The frames undergo processing via VGG19, which is pre-trained on ImageNet, to extract spatial features, yielding $\sigma_{m_{F}}\leq \frac{1}{3}\delta_{1}$ feature maps for each frame. Audio inputs *A*_*t*_ are processed using a source separation module (ConvTasNet) and subsequently analyzed by VGGish, which is pre-trained on AudioSet, to extract 128-dimensional acoustic features that encapsulate aural patterns. An audio-guided attention mechanism is utilized to align and augment visual aspects in accordance with aural cues. The polished visual and unprocessed audio characteristics are input into distinct LSTMs to model temporal dependencies, thereby capturing sequential patterns in the modalities. The outputs of the LSTMs are integrated within the DMRN, which improves modality interaction by concurrently updating and maintaining complementary information. The integrated representation is ultimately processed through a fully linked network for classification, accomplishing efficient multimodal integration and temporal alignment to enhance robust audio-visual event localization.

### 3.3 Training

The proposed model was trained using the Audio-Visual Event (AVE) dataset, comprising temporally labeled audio-visual events across 28 categories. The model design incorporates a Dual Multimodal Residual Network (DMRN), Long Short-Term Memory (LSTM) networks, and audio-guided attention processes. The training procedure aimed to enhance the synchronization and integration of auditory and visual data for accurate event localization.

The audio-guided attention layer was trained to selectively concentrate on spatial areas in the visual feature maps that align with the auditory items in the audio stream. Visual features, obtained from a pre-trained VGG19 network, were input into the attention layer in conjunction with auditory information from the VGGish model. The attention mechanism calculates a weighted sum of visual elements, influenced by auditory inputs, to produce attended visual features. The attention weights are acquired during the back propagation process, allowing the model to dynamically synchronize visual focus with aural signals over time.

The LSTM networks were employed for the temporal modeling of the attended visual and audio aspects. Distinct LSTM networks were utilized for each modality to capture the temporal dependencies in audio and visual streams. The LSTM received a sequence of attended visual features as input for visual data, but the input for audio data consisted of the raw feature vector recovered by VGGish. Each LSTM contained 128 hidden units, and its outputs were processed via the DMRN for multimodal fusion. Gradients for the LSTM were calculated via Back Propagation Through Time (BPTT), allowing the model to acquire both short-term and long-term dependencies within each modality.

The DMRN amalgamated the outputs of the LSTM networks to generate a unified multimodal representation. The residual network utilized an additive fusion function to amalgamate complementing information from the auditory and visual streams while maintaining modality-specific characteristics. This approach improved the integrated feature representation, enabling effective classification of audio-visual events. The network utilized a solitary residual block, as supplementary blocks did not enhance performance during empirical assessment. The integrated representation was processed through fully connected layers with a softmax activation function to forecast the event category for each video clip.

The complete model, comprising the attention layer, LSTM, and DMRN, was trained in an end-to-end manner with the Adam optimizer. The initial learning rate was established at 0.001, employing a step decay schedule that decreases the learning rate by a factor of 0.1 every 15,000 steps to enhance convergence. The training procedure utilized a batch size of 64 for a total of 300 epochs. The model was refined utilizing a Multi-Label Soft Margin Loss algorithm, which guaranteed precise categorization across various categories.

Regularization methods, including dropout with a probability of 0.5, were implemented in the fully connected layers to mitigate overfitting. L2 regularization was applied to all trainable parameters. Early stopping was utilized based on validation loss, with checkpoints preserved for the optimal model.

The AVE dataset was divided into training, validation, and test subsets. Validation occurred every five epochs, and the optimal model was chosen based on validation accuracy. During evaluation, the model’s predictions were compared to the actual labels for temporal event localization. The assessment criterion employed was overall accuracy, illustrating the model’s efficacy in utilizing attention mechanisms, temporal dependencies, and multimodal fusion for effective event localization.

### 3.4 Performance evaluation of audio source separation module

The WSJ0-2mix [26] dataset serves as a benchmark for audio source separation, created by dynamically mixing clean utterances from the WSJ0 corpus with overlapping speech from two speakers at varying signal-to-noise ratios (SNRs). Models like Conv-TasNet and DPRNN-TasNet are trained using this dataset and evaluated on unseen test sets with metrics such as SI-SNRi and SDRi, which measure improvements in separating overlapping speech while maintaining audio quality.

In this table, the “±” numbers denote the standard deviation, reflecting the variability of the performance measures among various test samples. The metrics indicate that Conv-TasNet excels in noise separation tasks, as shown by elevated SI-SNRi and SDRi values. DPRNN-TasNet demonstrates enhanced efficacy in human speech separation, attaining elevated SI-SNRi and SDRi scores, signifying its proficiency in speech-centric tasks. Following training, the performance metrics of the DPRNN-TasNet model showed significant improvements. As illustrated in Table 1.

**Table 1:** Performance evaluation for ConvTasNet and DPRNNTasNet on WSJ0-2mix and WSJ0-3mix dataset.

Model	Dataset	SI-SNRi (dB)	SDRi (dB)
Conv-TasNet-gLN	WSJ0-2mix	14.9 ± 0.3	15.1 ± 0.2
Conv-TasNet-cLN	WSJ0-2mix	10.1 ± 0.4	10.7 ± 0.3
DPRNN-TasNet	WSJ0-2mix	18.6 ± 0.2	18.7 ± 0.2
Conv-TasNet-gLN	WSJ0-3mix	12.4 ± 0.3	12.7 ± 0.3
Conv-TasNet-cLN	WSJ0-3mix	7.3 ± 0.4	7.7 ± 0.4

Global Layer normalizing (gLN) and Cumulative Layer Normalization (cLN) in Conv-TasNet utilize distinct normalizing techniques to stabilize network training and improve performance. gLN concurrently normalizes all features, effectively capturing global dependencies, as demonstrated by its superior performance metrics in both two-speaker (14.9 dB SI-SNR) and three-speaker (12.4 dB SI-SNR) mixing scenarios in comparison to cLN. In contrast, cLN separately normalizes each feature over time, allowing for dynamic changes within individual features, while yielding marginally lower performance scores (10.1 dB and 7.3 dB SI-SNR for two and three-speaker mix scenarios, respectively). This demonstrates the effect of normalization methods on managing diverse audio complexity in speaker separation task.

For background chatter, models like DPRNN-TasNet achieve superior results, with SI-SNRi and SDRi of 18.6 dB and 18.7 dB, respectively, on the WSJ0-2mix dataset, effectively handling overlapping speech. Conv-TasNet-gLN also performs well in multi-speaker scenarios, achieving 14.9 dB SI-SNRi on WSJ0-2mix and 12.4 dB on WSJ0-3mix, showcasing its capability in dynamic noise environments. However, mechanical noises prove more challenging, which showed lower performance in Conv-TasNet-cLN, respectively. This variation highlights the system’s capacity to maintain intelligibility and separation quality across diverse noise profiles.

The mir_eval library offers an extensive collection of evaluation metrics crucial for music information retrieval and audio signal processing. This library facilitates the implementation of metrics such as Source to Interference Ratio Improvement (SI-SNRi) and Signal to Distortion Ratio Improvement (SDRi), which are pivotal for evaluating the effectiveness of audio separation and enhancement algorithms. Utilizing mir_eval [27], referenced in, ensures that the performance assessment of audio processing tasks is both reproducible and comparable to other research, making it a standard tool in both academic and commercial research projects.

**Fig 5:**
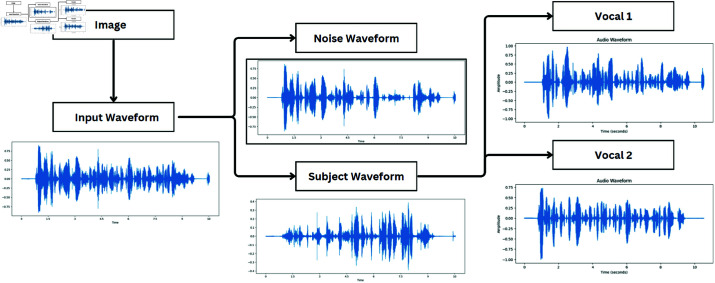
Audio and visual source separation waveforms-1.

**Fig 6:**
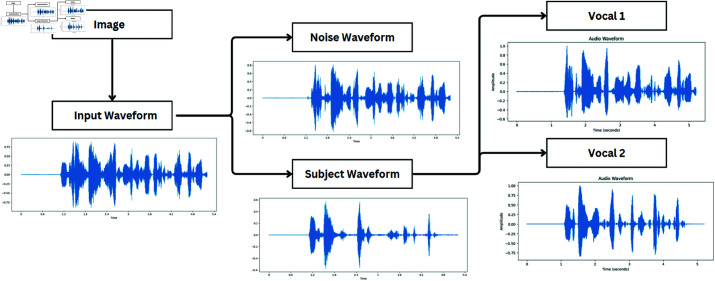
Audio and visual source separation waveforms-2.

Figs 5 and 6 shows the noise and human voice separation output of convTasNet noise output and DPRNNTasNet human voice separated output of trained model. The model accurately separate the human voice from the input data with better audio quality

### 3.5 Accuracy of audio-visual event localization

**Fig 7:**
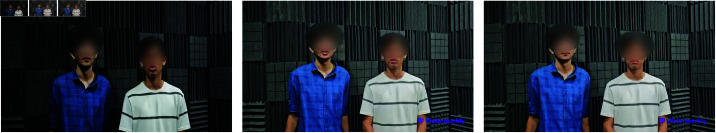
Human speech video frame.

**Fig 8:**
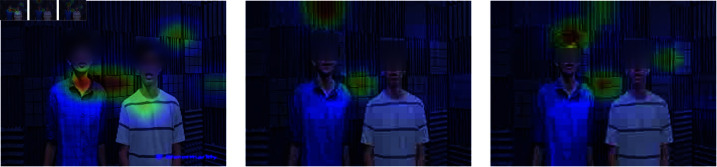
Attention heat map on video frames.

Figs 7 and 8 illustrate the process flow and heat maps used to delineate areas within video frames where sounds originate, particularly in scenes crowded with multiple individuals. The heatmaps, derived from the “affine h” layer using a forward hook technique, are superimposed onto the video frames with a color-mapping technique to visually highlight the regions corresponding to distinct audio sources. Enhanced visual contexts provided in Figs 7 and 8 through graphic heatmaps assist in accurately identifying audio sources. This integration of auditory and visual signals significantly enhances the model’s ability to isolate and identify multiple sound sources in complex environments, thereby improving precision and clarity in localizing event sounds[28–30] amidst various background noises [31].

**Fig 9:**
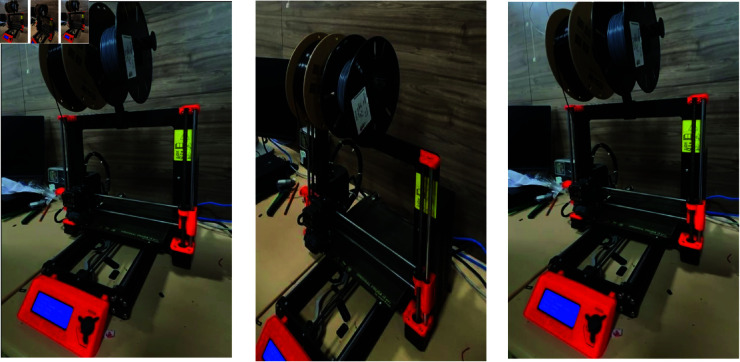
Non-human sounds video frames.

**Fig 10:**
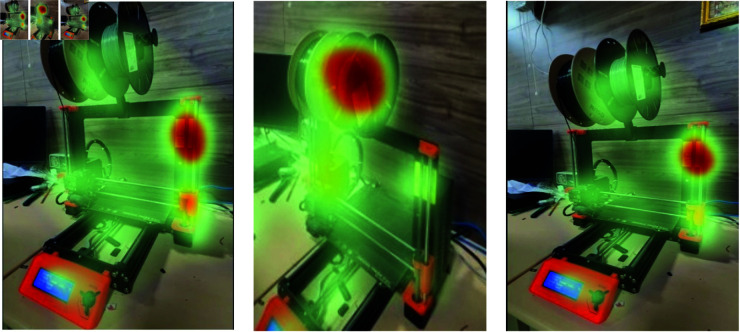
Attention heat map on non-human video frames.

Fig 9 shows the video frames of a 3D printer, together with sound waveforms and heat maps indicating the location of the sound in the Fig 10. This demonstrates the system’s capacity to precisely determine the location of sound sources that are not produced by humans within the visual frames. In the end, after getting the heat maps on video frames and separated audio sources, audio sources can be then remixed according to the needs. In order to perform this operation, a Python package called pydub is used to adjust the amplitude of the audio files and then knit them together with the video frames using ffmpeg. Thus, the desired output of audio source localization with control over audio sources is obtained.

Table 2 presents a comparative comparison of the Audio-Guided Visual Attention (AGVA) model and the Dual Multimodal Residual Network (DMRN). AGVA achieves a training accuracy of 71.24% and a testing accuracy of 68. 0%, while DMRN surpasses it with a training accuracy of 72.92% and a testing accuracy of 71.88%. This enhancement is due to DMRN’s use of modern audio and visual weights, further augmented by LSTMs to capture temporal interdependence. The amalgamation of auditory and visual elements in DMRN enhances synchronization and contextual comprehension, hence augmenting localization precision for audio sources. The results demonstrate DMRN’s superior capability in managing intricate situations, such as overlapping audio occurrences, in contrast to AGVA. This signifies that DMRN is a more resilient and reliable framework for tasks related to audio-visual source separation and localization. The results reveal that DMRN outperforms others due to the improved audio and visual weights facilitated by LSTMs.

**Table 2:** Comparison analysis of audio-guided visual attention and dual multimodal residual network.

Model	Train Accuracy	Test Accuracy
Audio-Guided Visual Attention	71.24	68.08
Dual multimodal residual network	72.92	71.88

### 3.6 Ablation study

A comprehensive ablation study is conducted, as illustrated in Table 3, to assess the effects of various modalities, attention processes, and degrees of supervision. This analysis examines the performance contributions of each model configuration, providing insight into the importance of auditory, visual, and multimodal aspects. The notations in the table are defined as follows: **A** denotes models utilizing audio-exclusive attributes, whereas **V** signifies models employing visual-exclusive attributes. **V-att** pertains to visual attributes augmented by the audio-directed attention technique. **A+V** integrates audio and visual elements without attention, but **A+V-att** utilizes attended features from both modalities, exemplifying the comprehensive supervised methodology. Weakly-supervised models, indicated by a **W-** prefix, utilize audio-only features in **W-A** and visual-only features in **W-V**. **W-V-att** amalgamates visual attributes with attention, whereas **W-A+V** and **W-A+V-att** synthesize audio-visual attributes without and with attention, respectively.

**Table 3:** Event localization prediction accuracy (%).

Model	A	V	V-att	A+V	A+V-att	W-A	W-V	W-V-att	W-A+V	W-A+V-att
Accuracy	61.2	57.9	60.9	68.08	71.8	55.2	56.1	57.9	66.7	68.9

The results demonstrate several significant findings. Initially, the incorporation of audio and visual elements markedly improves performance, as seen by the **A+V** model attaining an accuracy of 68.08%, in contrast to 59.5% for the audio-only model and 55.3% for the visual-only model. The use of audio-guided attention enhances performance, as evidenced by the **A+V-att** model, which attains the maximum accuracy of 71.8%, illustrating the efficacy of attention mechanisms in aligning and refining multimodal elements.

Attention mechanisms enhance performance in single modalities as well. The **V-att** model demonstrates an accuracy enhancement to 58.6%, in contrast to the baseline **V** model at 55.3%, underscoring the efficacy of auditory direction in augmenting visual feature significance. Likewise, weakly-supervised models demonstrate strong performance in the absence of explicit temporal annotations, with **W-A+V-att** attaining 66.7%, highlighting the robustness of the proposed method in less restrictive conditions.

This ablation study confirms the significance of integrating modalities and utilizing attention mechanisms. The incremental advancements in models illustrate the benefits of multimodal integration and attention in improving event localization precision, even with limited supervision. These findings underscore the importance of attended audio-visual characteristics as a fundamental element of the proposed architecture.

### 3.7 Comparison with existing methods

Table 4 shows the performance comparisons between our method and state-of-the-art methods in terms of both fully- and weakly-supervised AVE localization tasks under the fair experimental setting. Our method follows the Dual Multimodal Residual Network and consistently achieves the high accuracies, i.e., 72.92% and 71.88%, outperforming all other methods, which only adopt one direction. The rich high level semantic audio-visual features extracted from our Multi-modality method provide exact predictions.

**Table 4:** Comparisons between our method and state-of-the-art methods.

state-of-the-art methods	Test Accuracy
Yapeng *et al*. [4]	55.3
Yu-Chiang *et al*. [27]	72.60
Zhou *et al*. [1]	57.0
Zhang *et al*. [17]	63.07
Our proposed method	72.92

The suggested methodology for audio-visual source separation is characterized by its advanced integration of deep learning models, including ConvTasNet and DPRNNTasNet, which effectively address intricate audio situations, such as overlapping speech. The implementation of a Deep Multi-Resolution Network (DMRN) for audio-visual fusion guarantees accurate localization and management of audio sources, hence improving user engagement in video conferences. The individual control over audio sources enables dynamic adjustments of volume and clarity for each participant, hence enhancing communication efficiency and user experience in multi-speaker settings.

## 4 Conclusion

This work proposes a novel pipelined architecture designed to enhance audio-visual interactions in multi-presenter settings such as video conferences. Our framework leverages deep learning-based audio and video feature extractors, DMRN for multi-modality signal fusion, and DPRNNTasNet for human voice separation. Additionally, the model’s audio-visual event localization was evaluated with 100 test samples, scoring an average accuracy of 72.92 for localizing audio sources. Experimental results demonstrate that our framework effectively detects and distinguishes speakers, while allowing for individual volume adjustments. This feature enhances clarity and user experience by dynamically categorizing and controlling audio events in videos. Our methodology addresses significant challenges in video communication, resulting in substantial improvements in user engagement and communication efficiency. The architecture provides an advanced solution for complex multi-speaker scenarios by accurately determining speaker locations and effectively managing sound. This approach promises to elevate the quality of virtual communication across various platforms, offering a robust foundation for future advancements in audio-visual technology. This framework facilitates further investigation into real-time processing capabilities and scalability to accommodate larger datasets and more intricate scenarios, potentially extending to practical applications such as augmented and virtual reality systems where accurate audio-visual synchronization is essential.
